# Clinical Presentation of Myocarditis in the Pediatric Age Group and Predictors of Poor Early and Late Outcomes: Academic Hospital Experience

**DOI:** 10.7759/cureus.31643

**Published:** 2022-11-18

**Authors:** Aliaa S Alamri, Luay T Khayat, Ahad J Alzahrani, Lujain K Kurdi, Najwa F Alkhameesi, Saud A Bahaidarah

**Affiliations:** 1 Pediatrics, Faculty of Medicine, King Abdulaziz University, Jeddah, SAU; 2 Pediatrics, Faculty of Medicine, King Abdulaziz University Hospital, Jeddah, SAU

**Keywords:** pediatric clinical cardiology, saudi arabia, outcomes, children, myocarditis

## Abstract

Background: Myocarditis is a leading cause of morbidity and mortality in the pediatric age group and contributes to a wide range of complications, including dilated cardiomyopathy, congestive heart failure, and even death, so early identification and comprehensive management are essential for a favorable outcome.

Objectives: Summarize the presenting clinical signs and symptoms of pediatric patients with a diagnosis of myocarditis and a poor outcome and correlate the clinical presentation and laboratory and radiographic findings to identify possible predictors of a poor outcome.

Methods: This retrospective cohort study included all patients who were diagnosed with myocarditis and followed up at King Abdulaziz University Hospital, Jeddah, Saudi Arabia over the 13 years between January 01, 2007, and December 31, 2019. Information on patient demographics, clinical presentation, and non-invasive investigations was obtained. Poor outcomes were defined as death or evidence of left ventricular dysfunction on echocardiography which was evaluated at two points in time as an early outcome and a late outcome.

Results: Seventeen patients (male 52.9%, female 47.1%) with a median age of 4 ± 4.31 years are included. The most frequent initial complaints were exercise or feeding intolerance, respiratory symptoms, and shortness of breath. On the other hand, hepatomegaly and respiratory distress were the most common clinical signs. All the patients were admitted to the pediatric intensive care unit but only 41% required mechanical ventilation. The presence of a murmur at presentation was significantly correlated with a poor early outcome. Ischemic changes on the electrocardiogram and moderate left ventricular dysfunction on the echocardiogram were significantly correlated with a poor late outcome.

Conclusion: Diagnosis of myocarditis can be established by a combination of clinical presentation and investigative tools. A murmur, ischemic changes on the electrocardiogram, and left ventricular dysfunction are important predictors of myocarditis in children.

## Introduction

Myocarditis is an important cause of morbidity and mortality in the pediatric age group. It is defined as an inflammatory condition of the myocardium, with infiltration of leukocytes followed by necrosis and fibrosis of myocytes [1-2]. Although myocarditis has multiple causes, it is most commonly implicated in viral infections, which lead to the activation of the immune system and injury to the myocardium. Non-infective causes, such as toxins, hypersensitivity reactions, and autoimmune processes, are less common [2-5].

The exact incidence of myocarditis is unclear due to underdiagnosis. Studies have shown that myocarditis-related admissions comprise 0.02%-0.05% of all medical pediatric and pediatric surgery ward admissions and that there is a male predominance (77%) after the age of seven years [1, 6].

Myocarditis has a triphasic nature and can be classified clinically as acute, fulminant, or chronic. The presentation of acute myocarditis ranges from nonspecific symptoms (fever, myalgia, palpitations, or exertional dyspnea) to fulminant hemodynamic collapse and sudden death [6] and has a reported mortality rate of 7%-15% in the acute phase [2, 7].

A study of pediatric patients with myocarditis found that 83% of cases were not diagnosed at the time of the first presentation to the physician and required two or more visits to a medical provider before suspicion was raised for myocarditis [1]. In some cases, the initial presentation is sudden unexplained death [1, 5, 8]. Although mild cases of myocarditis may go unrecognized by the healthcare provider, many cases of myocarditis are misdiagnosed as other mild illnesses before they deteriorate further to severe illness [6, 8].

Myocarditis is an important precursor of dilated cardiomyopathy and left ventricular dysfunction [3]. Long-term follow-up over a mean of three years in patients diagnosed with acute myocarditis showed that up to 21% developed dilated cardiomyopathy [9]. However, it has been recognized that 9% of cases of dilated cardiomyopathy are caused by myocarditis [7] and that dilated cardiomyopathy is the leading indication for heart transplantation [10]. Other sequelae of myocarditis include congestive heart failure, ventricular arrhythmias, and sudden death [11]. Although some patients recover with simple supportive care, others require mechanical ventilation, hemodynamic support, intensive care admission, and heart transplantation [4, 11].

The aims of this study are to (1) summarize the presenting clinical signs and symptoms of pediatric patients at King Abdulaziz University Hospital with a diagnosis of myocarditis and a poor outcome, such as dilated cardiomyopathy, congestive heart failure, arrhythmia, or sudden death in hospital or during follow-up from 2007 to 2019 and (2) correlate the clinical presentation and laboratory and radiographic findings to identify possible predictors of a poor outcome.

## Materials and methods

Patient selection

This hospital-based, retrospective cohort study was approved by the Research and Ethics Committee of King Abdulaziz University Hospital, Jeddah, Saudi Arabia, and included all patients who were diagnosed with myocarditis and followed up at King Abdulaziz University Hospital between January 01, 2007, and December 31, 2019. The diagnosis was based on the International Classification of Diseases, Tenth Revision, and was made by pediatric cardiology consultants or specialists [12]. The exclusion criteria were as follows: acute neonatal myocarditis, underlying collagen vascular disease, secondary cardiomyopathy (Kawasaki disease, neuromuscular disorders, cardiotoxic agents, an inborn error of metabolism, familial syndromes, malformation syndromes, or sepsis), coronary artery anomalies, congenital heart disease, chronic bacterial sepsis, chronic primitive arrhythmia; patients who underwent surgery were also excluded.

Data collection

The study data were collected from the hospital information system. Inpatient data were defined as those obtained from the time the patient was admitted as an emergency with clinical symptoms suspicious of acute myocarditis. Outpatient follow-up data were obtained for up to one year following hospitalization. Information on patient demographics and characteristics, symptoms at the time of diagnosis, findings on physical examination, diagnostic laboratory and imaging results, treatment given, and outcomes data were also obtained.

Definitions

Myocarditis

Myocarditis was categorized as probable acute myocarditis or possible subclinical myocarditis depending on the presence of cardiovascular symptoms and at least one of the following: elevated biomarkers; electrocardiogram (ECG) [4, 13-14]; echocardiographic or cardiac MRI [15]; and definitive evidence of inflammation on cardiac MRIs (late gadolinium enhancement sequence) [16]. 

Cardiac Syndrome

Myocarditis is classified into four different cardiac syndromes based on their clinical presentation: (1) dysrhythmia (symptoms of palpitations associated with an abnormal rhythm on the ECG) [13]; (2) congestive heart failure without hemodynamic instability; (3) fulminant myocarditis (sudden cardiac death or abrupt cardiogenic shock); and (4) acute coronary syndrome-like symptoms of chest pain, abnormal ECG findings suggestive of myocardial ischemia, and an increased troponin T level [13, 17]. Abnormal laboratory measurements were defined as raised troponin T (>0.04 µg/L) and C-reactive protein (>3 mg/L) levels [2].

Outcomes

Outcomes of myocarditis were categorized as early or late. Poor early outcomes were defined as death or evidence of left ventricular dysfunction on echocardiography (ejection fraction <40%) at the time of hospital discharge. Poor late outcomes were defined in the same way but after one year of follow-up. Risk factors associated with a poor early or late outcome were evaluated in our cohort study.

Statistical Analysis

Categorical variables are shown as the frequency and percentage while quantitative variables as the mean and standard deviation were calculated with a 95% confidence level. The regrouping of variables was done based on the study objectives. All statistical analyses were performed using the SPSS for Windows version 21 (IBM Corp., Armonk, NY, USA). A p-value <0.05 was considered statistically significant.

## Results

Seventeen patients diagnosed and treated between January 2007 and December 2019 met our inclusion criteria. The median age at the time of the diagnosis was 4 ± 4.31 years. The study population contained more males than females [52.9% (9/17) vs 47.1% (8/17)]. Only 29.4% (5/17) were Saudis and 70.6% (12/17) were non-Saudis. Further demographic data are shown in Table 1.

**Table 1:** Demographic data. SD, standard deviation

Variables	Number (%)
Age at diagnosis	
Mean ± SD	4 ± 4.31
Gender	
Male	9 (52.9% )
Female	8 (47.1%)
Ethnicity	
Saudi	5 (29.4%)
Non-Saudi	12 (70.6%)

Clinical presentation of myocarditis

In most cases (76.5%, 13/17), the final diagnosis of myocarditis was made during the first visit to our emergency department (ED); (17.6%, 3/17) required two ED visits before the diagnosis of myocarditis was made. The majority of patients (76.5%, 13/17) presented with congestive heart failure without hemodynamic instability; (11.8%, 2/17) presented with fulminant myocarditis (Table 2). All patients needed admission to the pediatric intensive care unit before their final diagnosis.

**Table 2:** Clinical presentation. ED, emergency department; ICU, intensive care unit

Variables	Number (%)
ED visit before the diagnosis of myocarditis	
One visit	13 (76.5%)
Two visits	3 (17.6%)
More than two visits	0 (0%)
No visits	1 (5.88%)
Pediatric ICU admission	
Yes	17 (100%)
No	0 (0%)

There was some overlapping of symptoms and signs at the first presentation (Table 3). The most common cardiac symptom was exercise or feeding intolerance (76.5%, 13/17), followed by shortness of breath (58.8%, 10/17). The most common non-cardiac symptoms were respiratory-related (64.7%, 11/17). 

**Table 3:** Poor early and late outcomes and cardiac syndromes.

Variables	Number (%)
Poor early outcome	
Persistent left ventricular systolic dysfunction	10 (58.8%)
No disease (cured)	2 (11.8%)
Death	5 (29.4%)
Poor late outcome	
Persistent left ventricular systolic dysfunction	3 (17.6%)
Recovered	8 (47.1%)
Death	1 (5.9%)
Irrelevant (died early)	5 (29.4%)
Cardiac syndrome	
Congestive heart failure	13 (76.5%)
Fulminant myocarditis	2 (11.8%)
Dysrhythmia	2 (11.8%)
Acute coronary syndrome	0 (0%)

In children with signs of heart failure, hepatomegaly and respiratory distress were the most common cardiac signs at the time of initial presentation (82.4%, 14/17). Other signs of heart failure are shown in Table 3.

Diagnostic investigations

Details of the specific laboratory investigations together with chest radiograph, ECG, and echocardiography performed in the patients enrolled in this study are shown in Table 4. C-reactive protein was measured in all patients as an indicator of the systemic inflammatory response and was found to have a mean value of 22.46 mg/dL. The plasma level of troponin T, a biomarker for myocardial injury, was assessed in most patients and had a mean value of 6.83 µg/L.

**Table 4:** Diagnostic investigations and treatment. CRP, C-reactive protein

Variables	Number (%)
Elevated CRP	15 (88.2%)
Elevated troponin T	15 (88.2%)
Chest X-ray	
Cardiomegaly	9 (52.9%)
Pulmonary edema	5 (29.4%)
Pulmonary infiltration	5 (29.4%)
Pleural effusion	5 (29.4%)
Electrocardiogram	
Sinus tachycardia	8 (47.1%)
Ischemic changes	3 (17.6%)
Prolonged QT interval	2 (11.8%)
Atrial tachycardia	2 (11.8%)
Premature ventricular complexes	1 (5.9%)
Low voltage	1 (5.9%)
Ventricular tachycardia	1 (5.9%)
Supraventricular tachycardia	0 (0%)
Echocardiography	
Mild left ventricular systolic dysfunction	3 (17.6%)
Moderate left ventricular systolic dysfunction	8 (47.1%)
Severe left ventricular systolic dysfunction	5 (29.4%)
Left ventricular dilatation	15 (88.2%)
Mitral valve regurgitation	2 (11.8%)
Biventricular systolic dysfunction	1 (5.9%)
Segmental wall motion abnormalities	1 (5.9%)
Treatment	
Diuretic 100% (17)	17(100%)
Digoxin 47.1% (8)	8 (47.1%)
Mechanical ventilation 41.2% (7)	7 (41.2%)
Spironolactone 41.2% (7)	7 (41.2%)
Angiotensin-converting enzyme inhibitor 23.5% (4)	4 (23.5%)
Beta-blocker 23.5% (4)	4 (23.5%)
Pacemaker 5.9% (1)	1 (5.9%)

Treatment and intervention

Almost all patients received cardiac medications during their hospital stay as listed in Table 3. All patients received diuretics. Mechanical ventilation was used in (41.2%, 7/17), and a pacemaker in (5.9%, 1/17).

Poor early and late outcomes and risk factors

Nearly all patients (88.2%, 15/17) had a poor early outcome and a small proportion had a poor late outcome (23.5%, 4/17). On discharge, (58.8%, 10/17) had persistent left ventricular systolic dysfunction and (29.4%, 5/17) died in the hospital. Poor late outcomes included persistent left ventricular systolic dysfunction (17.6%, 3/17), some of which were fatal (5.9%, 1/17). When all age groups were included, (47.1%, 8/17) made a full recovery from early left ventricular systolic dysfunction as a late outcome. However, (35.3%, 6/17) of patients despite different age groups died. The outcome of early left ventricular dysfunction was significantly correlated with the presence of a murmur (p=0.008). The outcome of late left ventricular dysfunction was strongly associated with ischemic changes on the ECG and moderate left ventricular dysfunction on the echocardiogram (p=0.014 and p=0.043, respectively).

## Discussion

We assessed retrospectively the clinical presentation of myocarditis, poor outcomes, and possible predictors of poor outcomes. Clinical presentation, laboratory workup, and certain imaging data were analyzed. There was a predominance of boys (52.9% vs 47.1%) but it is not known whether or not there is a true sex-related difference in the risk of myocarditis in children when compared with adults. However, a recent retrospective study in Finland found no specific sex-related difference in the risk of myocarditis in the first six years of life but found that the risk was higher in boys than in girls at the age of 6-15 years [3]. This age group coincides with hormonal changes and an increase in testosterone levels in boys [3]. And experiments in mice have confirmed that sex hormones played a major role in inducing myocarditis [18]. Furthermore, multiple studies had shown that the male sex is the predominant gender distribution compared to females. However, the predominance of boys in our study was not statistically significant.

A diagnosis of myocarditis can be elusive and challenging in children because of its diverse symptoms and clinical findings that are often unclear, nonspecific, and vary with age [16].

Clinical presentation of myocarditis can be vague in children and in this study feeding intolerance, shortness of breath, and respiratory tract symptoms were the common three presentations, and similarly, another retrospective study viewed shortness of breath, vomiting, and poor feeding as the most common presenting symptoms [13]. Although other studies had reported respiratory symptoms as the most common presenting symptom, and some stated gastrointestinal symptoms as the most common [1, 17, 19-20]. Moreover, in a study by Sankar et al., 10 children had a diagnosis of acute fulminant myocarditis, and the most common clinical presentations were fever, breathing difficulty, and body swelling [21]. This diversity of symptoms can best be explained by differences in age in that unlike younger children and infants, older children can display adult-like symptoms. However, the etiology of myocarditis may play a role in the wide spectrum of clinical presentations of myocarditis. 

The most common cause of myocarditis is believed to be viruses, especially enteroviral viruses. Remarkably, 58.8% of our patients had previous viral conditions, which is consistent with other studies [5, 20]. In a study of the relationship between symptoms and type of virus by Mahrholdt et al. in Germany, patients who were infected with human herpesvirus six infections displayed signs and symptoms of congestive heart failure while other patients who were infected with parvovirus B19 had chest pain mimicking acute myocardial infarction but without any evidence of coronary artery disease [22].

Most of our patients with clinical evidence of heart failure presented with hepatomegaly and respiratory distress, which is consistent with a report by Durani et al. [13]. However, other studies have found that tachycardia and tachypnea are the most significant cardiac signs [5, 8, 20, 23]. Therefore, detecting myocarditis in children based on their clinical presentation is challenging [20].

In our study, the majority (76.5%) of patients were diagnosed with myocarditis on the first visit to the ED and only 17.6% made two ED visits before the diagnosis was made. However, in another study, 59% of patients needed two or more ED visits and only 41% were diagnosed during the first visit [20]. This variant might be due to the inaccessibility of pediatric echocardiography services at their center.

All patients in our study required admission to the pediatric intensive care unit and 80.4% of those in a US study were admitted to the intensive care unit from 2006 to 2011 [15]. Therefore, it is crucial to take rapid action and arrange admission to the pediatric intensive care unit for assessment and stabilization.

 The diagnostic accuracy of most non-invasive tests for myocarditis is another reason for the difficulty encountered when diagnosing this illness. We observed elevated C-reactive protein levels in 88.2% of our study population, whereas, in another study, only 16% of patients had an elevated C-reactive protein level [20]. Although the C-reactive protein level can be elevated in the acute phase, it is neither sensitive nor specific for detecting active myocardial inflammation [16]. Therefore, normal results do not exclude an inflammatory process in the myocardium. Moreover, 88.2% of our study population had an elevated troponin T level. Although elevated troponin T can support a clinical suspicion of myocarditis in children, a normal result does not rule out myocarditis [16].

Myocarditis is almost always characterized by an abnormal ECG in children [7]. An abnormal ECG identified sinus tachycardia in 46.1% of our cases and ischemic changes were present in 17.6% of cases. Furthermore, several abnormalities show wide variation, and no specific abnormality is sufficient to serve as a marker [24]. A finding of cardiomegaly on a chest radiograph may lead to a diagnosis of cardiovascular disease, especially myocarditis, and exclude other differential diagnoses.

Echocardiography remains the most useful diagnostic test when myocarditis is suspected clinically [25]. Myocarditis is characterized by dilatation of the left ventricular and impaired global left ventricular dysfunction ejection fraction. In our study, the majority of children with myocarditis presented with some echocardiographic abnormalities on admission. Left ventricular systolic dysfunction was classified as mild in 17.6%, moderate in 47.1%, and severe in 29.4%. Abnormal segmental wall motion on echocardiography may also assist in diagnosing ischemic cardiomyopathy [16]. However, the availability of echocardiography in the ED is limited and requires considerable expertise and training. We believe that the above clinical and biochemical factors may be useful in identifying patients at high risk. Therefore, clinical presentation, radiographic imaging, and other investigation modalities can lead to a high index of suspicion for a diagnosis of myocarditis. Our findings highlight the accuracy and importance of certain investigations and the impact they have on the diagnosis of myocarditis, which may be missed if the clinician relies on the clinical presentation alone.

We found the most common syndrome associated with myocarditis was congestive heart failure, which was present in 76.5% of the children and manifested most commonly as hepatomegaly. By contrast, Rodriguez-Gonzalez et al. found that the most common presentation (in 34%) was acute coronary syndrome-like, followed by fulminant myocarditis and congestive heart failure in 29% and 23%, respectively [20]. However, most of their cases were younger than two years of age and 35% were older than 12 years, whereas the median age in our study was 4 ± 4.31 years with no specific bimodal age distribution [20].

There is a significant etiologic relationship between congestive heart failure and myocarditis, which often causes dilated cardiomyopathy resulting in a need for heart transplantation and even death. Most of our patients presented with congestive heart failure and all needed treatment with a diuretic as part of the treatment plan in the hospital.

Dysrhythmias are common in pediatric patients with myocarditis and are associated with poor outcomes. Ventricular arrhythmias were the most common type of arrhythmia in a study that included 85 patients with myocarditis who were aged younger than 21 years [26].

Clinical symptoms of palpitations and syncope have been recognized as part of the syndrome of dysrhythmia in children with myocarditis [5, 24]. Two of our patients had dysrhythmia but only one required the insertion of a pacemaker. Rodriguez-Gonzalez et al. similarly found that only one of 42 children with dysrhythmias required a pacemaker [20]. In another retrospective study that included nine pediatric patients diagnosed with myocarditis and complete atrioventricular block, it was found that heart function reverted to normal in most cases but that patients with low cardiac output after pacemaker insertion had a poor outcome and were at increased risk for mortality [27]. In conclusion, various types of arrhythmias have been reported, but most of them are resolved eventually [24]. However, cardiac monitoring is important in patients with myocarditis for early recognition and treatment of arrhythmias and the need for pacemaker insertion.

Myocarditis is a precursor to dilated cardiomyopathy and left ventricular dysfunction [6]. Poor early outcomes were detected in 88.2% of our patients and 23.5% of these patients had a poor late outcome.

Evaluating for poor early outcomes, left ventricular systolic dysfunction was found in 58.8% of our patients, 29.4% of whom died. Other researchers found that 50% of patients had left ventricular dysfunction on admission and a 5% mortality rate during hospitalization, which they attributed to the ability of myocarditis to generate significant alterations in the early stages of the disease [20]. Another study assessed the short-term outcomes of acute fulminant myocarditis in 10 children; one child died and five continued to have left ventricular dysfunction with valvular insufficiency [21] difference in mortality between our study and other studies might be attributed to multiple factors, but most important is probably the small number of patients with myocarditis at our center.

In univariate analysis, we identified some characteristics that could help clinicians to identify these patients on admission. We found a significant correlation between early left ventricular dysfunction and the presence of a murmur; the main pathophysiologic process is myocardial necrosis with interstitial edema which diminishes the contractile performance of cardiac muscle [16].

In this study, 23.5% of cases presented with a late outcome, 17.6% of whom had persistent left ventricular systolic dysfunction, which is strongly associated with ECG and echocardiographic findings.

The ECG findings are often abnormal in children with myocarditis but vary widely, ranging from sinus tachycardia to ST segment changes, including arrhythmias [5]. In this study, we observed a strong relationship between late poor outcomes and ECG abnormalities, in particular ischemic changes, which were found in 17.6% of patients with late left ventricular dysfunction. Therefore, changes in the ECG represent the effect of myocardial injury on cardiac function and thus the clinical outcome.

An earlier study also found a link between ECG findings and poor cardiac outcomes during long-term follow-up and when stratifying risk [24]. Therefore, given that ECG is so widely used, its features can be used to predict the late outcome in pediatric patients diagnosed with myocarditis.

This study has several limitations, including a retrospective single-center design with a small number of patients. A multicenter study might be needed to obtain better information. Our center lacks a heart transplant program. Therefore, the mortality rate in our study is higher than that in the recent literature. Finally, although our center used commonly available tools for the diagnosis of myocarditis, a more definitive diagnostic method, such as endomyocardial biopsy, is warranted.

## Conclusions

Myocarditis can be diagnosed by a combination of clinical presentation and investigation tools. A murmur, ischemic changes on the ECG, and left ventricular dysfunction are important predictors of myocarditis in the pediatric age group. All children should undergo echocardiography in view of its reliability in the diagnosis of myocarditis. Patients who presented with a significant murmur were at high risk of a poor early outcome. Ischemic changes on the ECG and moderate left ventricular dysfunction on an echocardiogram are predictors of a poor late outcome. Therefore, cardiac imaging tools are essential for the assessment of the severity and outcomes of myocarditis. These predictors can alert clinicians to the need for urgent early intervention with comprehensive management and follow-up.
